# Inflammatory Animal Model for Parkinson's Disease: The Intranigral Injection of LPS Induced the Inflammatory Process along with the Selective Degeneration of Nigrostriatal Dopaminergic Neurons

**DOI:** 10.5402/2011/476158

**Published:** 2011-04-17

**Authors:** A. Machado, A. J. Herrera, J. L. Venero, M. Santiago, R. M. de Pablos, R. F. Villarán, A. M. Espinosa-Oliva, S. Argüelles, M. Sarmiento, M. J. Delgado-Cortés, R. Mauriño, J. Cano

**Affiliations:** ^1^- Departmento de Bioquímica y Biología Molecular, Facultad de Farmacia, Universidad de Sevilla, 41012 Sevilla, Spain; ^2^- Instituto de Biomedicina de Sevilla (IBiS), Hospital Universitario Virgen del Rocío/CSIC, Universidad de Sevilla, 41013 Sevilla, Spain

## Abstract

We have developed an animal model of degeneration of the nigrostriatal dopaminergic neurons, the neuronal system involved in Parkinson's disease (PD). The implication of neuroinflammation on this disease was originally established in 1988, when the presence of activated microglia in the substantia nigra (SN) of parkinsonians was reported by McGeer et al. Neuroinflammation could be involved in the progression of the disease or even has more direct implications. We injected 2 *μ*g of the potent proinflammatory compound lipopolysaccharide (LPS) in different areas of the CNS, finding that SN displayed the highest inflammatory response and that dopaminergic (body) neurons showed a special and specific sensitivity to this process with the induction of selective dopaminergic degeneration. Neurodegeneration is induced by inflammation since it is prevented by anti-inflammatory compounds. The special sensitivity of dopaminergic neurons seems to be related to the endogenous dopaminergic content, since it is overcome by dopamine depletion. Compounds that activate microglia or induce inflammation have similar effects to LPS. This model suggest that inflammation is an important component of the degeneration of the nigrostriatal dopaminergic system, probably also in PD. Anti-inflammatory treatments could be useful to prevent or slow down the rate of dopaminergic degeneration in this disease.

## 1. Introduction

The pathological hallmark of Parkinson's disease (PD), an age-related neurodegenerative disorder, is the specific and progressive degeneration of dopaminergic neurons in the substantia nigra pars compacta (SNpc) [1]. This results in extrapiramidal motor dysfunction accompanied by progressive impairment of autonomy, mood, and cognitive functions [1–3]. Although some genes have been identified as responsible for rare familial early onset PD [4], its etiology remains elusive and many distinct possibilities have been pointed out for that. The effects of exogenous agents, infection, inflammatory response, and selective oxidative stress in the SN have been suggested as the most probable causes of the dopaminergic degeneration in the SN [5]. Many exogenous compounds have been described to produce degeneration of dopaminergic neurons in the nigrostriatal system, as 6-hydroxydopamine (6-OHDA), 1-methyl-4-phenyl-1,2,3,6-tetrahydropyridine (MPTP), rotenone and many other pesticides [6], which suggest an energetic metabolism impairment as main cause of the disease. Other possibilities were also proposed, as head trauma, encephalitis, reduced expression of trophic factors, alterations of the ubiquitin-proteasome system and neuroinflammatory mechanisms that are thought to collaborate in the progressive demise of SNpc neurons [1, 3, 7–13]. 

The implication of neuroinflammation as a component of the neurodegeneration progress of the disease was originally established in 1988 when the presence of activated microglia in the SN of PD was reported (see [14]; for review see [15, 16]). In that moment, the fact that many cases of PD were accompanied with a general brain inflammation along with the neurodegeneration was pointed out [14, 17, 18]. The dramatic proliferation of reactive amoeboid macrophages and microglia seen in the SN of parkinsonian brains, together with oxidative stress, was highly compatible with the existence of an inflammatory process similar to that previously described for Alzheimer's disease (AD) [19] and multiple sclerosis [20]. 

All these data allowed suggesting the implication of inflammation in the neurodegeneration of nigrostriatal dopaminergic neurons; however, its importance was not clear. Is this process exclusively involved in the progress of degeneration or could it have other implications? Is it able to induce the neurodegeneration of dopaminergic neurons?

To address these questions, we took advantage of lipopolysaccaride (LPS), a component of the Gram-negative bacteria cell wall. LPS is a potent inducer of inflammation and has diverse effects on cells of the immune system [21]. It was described that the intracerebral injection of LPS activated glial cells *in vivo* [22–26]; these microglial cells could contribute to the neurotoxicity by secreting neurotoxins [27–32] including inflammatory cytokines, N-methyl-D-aspartate (NMDA) receptor agonists, oxygen free radicals, and nitric oxide (NO). Besides, *in vitro* studies [33] showed that dopaminergic neurons were twice as sensitive to the toxic effects of LPS as tyrosine hydroxylase (TH) negative neurons. All these data suggested that the injection of LPS in different areas of the brain could be a good experimental model to ascertain the importance of inflammation in such different areas and neurons.

## 2. Nigral Dopaminergic Neurons Degeneration (Loss) Produced by the Inflammatory Processes Induced by the Intranigral Injection of LPS

**Figure 1:**
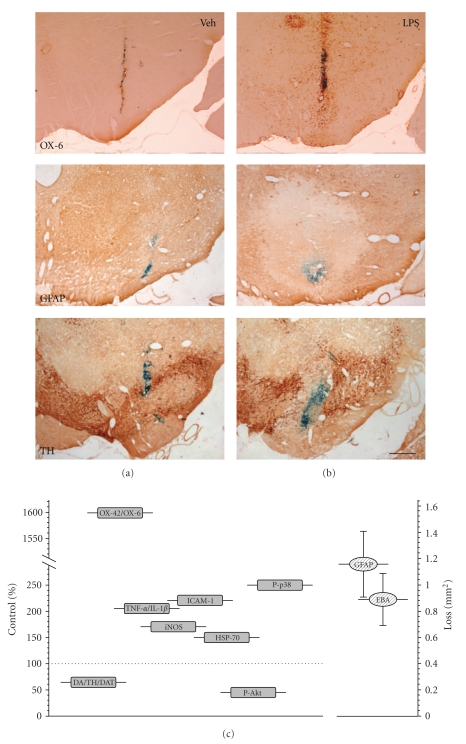
Effect of LPS on glial cells and dopaminergic neurons. (a) Injection of vehicle within the SN; (b) injection of LPS. OX-6 is a commercial antibody directed against a monomorphic determinant of the rat major histocompatibility complex (MHC) class II antigens, expressed by activated microglia but not for the resting cells. LPS increases OX-6 immunoreactivity around the injection track, filling the area of activated microglia characterized by its round morphology. On the contrary, there is an area lacking GFAP immunoreactivity, a marker of astroglia, around the injection site of LPS. As hallmark of this model, LPS induces the loss of dopaminergic (TH positive) neurons in the SN. Scale bar: 500 *μ*m. (c) Represents the average values of some parameters in the SN (as percentage of controls) after the single injection of 2 *μ*g of LPS: DA/TH/DAT, dopamine content, neurons expressing tyrosine hydroxylase, and dopamine transporter; OX-42/OX-6, density of activated microglial cells; amounts of the proinflammatory cytokines TNF-*α* and IL-1*β*, the adhesion molecule ICAM-1, the inducible nitric oxide synthase (iNOS), and the heat shock protein (HSP)-70; the phosphorylated (active) forms of the MAP kinases p38 (associated with promotion of apoptosis) and Akt (cell surviving signal). Alterations on the expression of GFAP and the endothelial barrier antigen (EBA), as area lacking expression (in mm_2_), are also shown.

We injected 2 *μ*L of a solution containing 1 mg of LPS/mL and 1% Monastral blue inert tracer in phosphate-buffered saline [34] in the left SN of female rats previously anesthetized. The results showed a significant inflammation with activation of microglia, a typical characteristic of brain inflammation. This effect was accompanied by the loss of dopaminergic neurons (Figure 1) and the decrease of the intracellular content of dopamine (DA) and its metabolites in SN and striatum. These effects suggested the loss of dopaminergic neurons of SN along with its terminals in striatum. This was corroborated by the loss of TH activity, mainly in striatum. The highest loss was found 15 days after the lesion and maintained during the 21 days of the experiments. The average loss of the dopaminergic system was around 35%; at this level, rats showed no obvious motor disturbances. Another interesting result came from serotonin (5-HT); although 5-HT levels in the SN reached a minimum 15 days after the lesion, they recovered to the initial levels by day 21. These results obviously suggested that the degenerative process induced by inflammation (LPS) in the SN was specific for dopaminergic neurons and allowed us to suggest that the injection of a single dose of LPS within the SN could be an interesting model for studying the selective effects of inflammatory reactions on dopaminergic system, and potentially for studying PD. Moreover, taking into account that Andersson et al. [23] had shown a loss of pyramidal cells in the CA1 field in mice following an acute intrahippocampal infusion of 2 *μ*g of LPS, we used this dose to study the specificity of the LPS effect in different areas and neurons of the brain. Specificity would obviously increase the importance of the effect. 

## 3. The SN Is the Most Sensitive Area of the Brain to the Inflammation Induced by the Injection of LPS, Which Produced a Selective Degeneration of Dopaminergic Neurons

We injected 2 *μ*L of the LPS solution described before into four different locations: the left SN, the medial forebrain bundle (MFB), the striatum, and the dorsal raphe nucleus (DR) [35]. LPS induced a strong macrophage/microglial reaction in SN, with a characteristic clustering of macrophage cells around blood vessels. These macrophage cells contained blue particles inside (from Monastral blue), indicating phagocytic activity. Fifteen days after injection, the number of lipid-laden cells began to decrease gradually and 1 year after injection most of the OX-42-positive cells showed ramified morphology. The SN was far more sensitive than the striatum and the other areas studied to the inflammatory stimulus. Moreover, only the dopaminergic neurons of the SN were affected, with no detectable damage to either the GABAergic or the serotoninergic neurons. The damage to the DA neurons in the SN was permanent, as observed 1 year after injection. Moreover, the injection of LPS into MFB did not produce any significant effect on the inflammation process, nor neuronal degeneration. Similar results were found when injections were made within the striatum or the DR. Therefore, these results seem to support that the injection of a single dose of LPS within the SN produced a selective effect on the dopaminergic neurons. Moreover, this work pointed out that inflammation was able to induce the degeneration of nigral dopaminergic neurons; the induction of inflammatory processes could be involved in the degeneration of dopaminergic neurons after injury (as boxing) or infection; it could also be involved in the progression of neuronal degeneration induced by other causes. Kim et al. [36] published the same conclusions. They also studied the effect of LPS injection within hippocampus and found no significant induction of inflammation or neuronal degeneration. Neurotoxicity after intraparenchymal injection of LPS has also been reported by other authors [37, 38].

## 4. Protective Effect Produced by the Treatment with Anti-Inflammatory Compounds on This Neurodegenerative Model

Obviously, the possible protective effect of an anti-inflammatory compound would support this model along with the importance of the inflammatory response in the LPS-induced degeneration of the nigral dopaminergic neurons. Inflammation is an attractive pharmacological target since it progresses over several days after injury and because intervention with anti-inflammatory agents may not result in intolerable side-effects [39]. 

### 4.1. Dexamethasone

Thus, we used dexamethasone, a potent anti-inflammatory drug. It was known that glucocorticoids were potent anti-inflammatory drugs that have long been used in clinical neurology for the treatment of brain inflammation [40, 41] and spinal cord injury [42]. They are potent inhibitors of the IFN-induced activation of microglia and macrophages *in vitro* [43] and downregulates the expression of the major histocompatibility complex (MHC) class II molecules on macrophages, both *in vivo* and *in vitro* [44]. Dexamethasone prevents the induction of cyclo-oxygenase (COX)-2 mRNA and prostaglandins in the lumbar spinal cord following intraplantar injection of Freund's complete adjuvant, in parallel with inhibition of edema [45]. Moreover, dexamethasone down-regulates the expression of MHC class II on rat microglia [43, 46] and reversibly inhibits the microglial proliferation *in vitro* [47] induced by axotomy and IFN-*γ*.

When animals were treated with dexamethasone [48], we found a significant protection against the damage caused by the intranigral injection of LPS. The treatment with dexamethasone interferes with many of the features characterizing proinflammatory glial activation, proliferation and also the upregulated expression of the MHC class II produced by LPS. We found that dexamethasone inhibited not only the number of OX-42-positive cells but also the number of microglia/macrophages expressing MHC class II antigens as revealed by staining with OX-6. These observations were in agreement with previous works reporting that glucocorticoids downregulate MHC class II [43, 44, 46]. 

Dexamethasone treatment was also able to prevent the loss of DA and its metabolites in SN and striatum. Similarly, it also prevented the loss of TH (measured by its activity and by TH immunostaining) in SN and striatum. These results confirmed that inflammatory response was implicated in LPS-induced neurodegeneration and open the possibility to use anti-inflammatory drugs to slow down the progress of the disease.

### 4.2. Minocycline

We have used other anti-inflammatory compounds; minocycline is a semisynthetic tetracycline derivative that is able to cross the BBB, reaching the cerebrospinal fluid [49]. Moreover, this new antibiotic exerts anti-inflammatory effects that are completely separated and distinct from its antimicrobial origin [50–52]; its use in neurodegenerative diseases has been suggested. Minocycline has been shown to protect hippocampal neurons against global ischemia [53] and to reduce cortical infarction volume in a rat model of focal brain ischemia [54]. It is also a very efficient neuroprotective agent in animal models of traumatic brain injury [55], multiple sclerosis [56], Huntington's disease [57], and amyotrophic lateral sclerosis [58]. Moreover, other studies had also demonstrated a neuroprotective effect of minocycline over the dopaminergic system in response to either MPTP [59, 60] or 6-OHDA [61]. 

When we evaluated the potential neuroprotective activity of minocycline in our animal model of Parkinson's disease induced by the intranigral injection of LPS [62], we found it was very effective preventing different inflammatory features along with the loss of nigral dopaminergic neurons. Minocycline treatment highly prevented the activation of reactive microglia as visualized by OX-42 and OX-6 immunohistochemistry, as well as the induction of interleukin (IL)-1*α* and tumor necrosis factor (TNF)-*α* mRNAs. Moreover, minocycline treatment also partially prevented the loss of dopaminergic neurons (12% against 50%) produced by LPS.

### 4.3. Simvastatin

At this time, some experimental and clinical evidence indicated that statins—extensively used in medical practice as effective lipid-lowering agents through the inhibition of 3-hydroxy-3-methylglutaryl-CoA reductase—had other cholesterol independent effects, as improving endothelial function, decreasing oxidative stress, inhibiting the thrombogenic response in the vascular wall, immunomodulatory and, anti-inflammatory properties [63–67]. Thus, we investigated the influence of simvastatin on the degenerative process of the dopaminergic neurons in our animal model of Parkinson's disease [68, 69]. In these works, we found that simvastatin treatment prevented the inflammatory process induced by LPS. Simvastatin prevented the bulk microglial activation found after LPS injection, reducing the number of microglia/macrophages expressing MHC class II antigens (40% of LPS group) as revealed by immunostaining with OX-6; it also inhibited the activation of IL-1*β*, TNF-*α*, and inducible nitric oxide synthase (iNOS) produced by LPS. These effects could be, at least, one of the causes of the prevention of dopaminergic neuronal loss induced by LPS produced by the simvastatin treatment. However, simvastatin showed another important effect that could help to the survival of dopaminergic neurons: simvastatin produced the activation of the neurotrophic factor BDNF, along with the prevention of the oxidative damage to proteins, conditions in which BDNF has an important protective effect. Moreover, it also prevents the main changes produced by LPS on different mitogen-activated protein kinases, featured as increases of P-c-Jun N-terminal protein kinase, P-extracellular signal-regulated kinase, p-38, and P-glycogen synthase kinase and the decrease of the promotion of cell survival signals such as cAMP response element-binding protein and Akt. Our results suggest that statins could delay the progression of dopaminergic degeneration in disorders involving inflammatory processes.

Many other anti-inflammatory compounds have been described to act as neuroprotectors in this and other models of Parkinson's disease. Nalaxone, an opiate receptor antagonist, and its inactive isomer (+)-nalaxone, inhibited both the activation of microglia and the loss of dopaminergic neurons induced by LPS in the SN [37, 38]. This effect was improved when nalaxone was combined with indomethacin [70]. Triptolide, another anti-inflammatory compound, also prevents the dopaminergic degeneration induced by LPS intranigral injection [71]. A similar effect was also produced by silymarin [72] and catalpol [73], other anti-inflammatory compounds.

## 5. Why Does the SN Have a Special Sensitivity to the Inflammation Induced by the Injection of LPS?

As described above, our results showed that the inflammatory reaction was stronger in SN than in striatum and other areas studied [34–36]. This was also clear in the characteristic disappearance of the GFAP immunoreactivity at the LPS injection site. We suggested at that moment that the differences could be due to specific structural differences between the SN and other areas. Regarding this, it was thought that SN had the highest concentration of microglia in the brain [74] and consequently a high concentration of the inflammation-related factors produced by these cells, such as TNF-*α* and NO. This proposal is supported by the fact that many anti-inflammatory compounds protect dopaminergic neurons. However, other authors have reported more recently that in intact brain, the densities of CD11b+ microglía are similar in SNpc and cortex, although LPS injection enhanced the number of CD11b+ cells in the former but not in the latter [75]. 

Other possibilities have been pointed out, as the increase in vascularization induced by inflammation in the SN, also described in a MPTP model of PD that is accompanied by the increase in the vascular endothelial growth factor (VEGF) [76]. One of the effects of the change could be the increase in the BBB permeability that could have some influence in the sensitivity, since the intranigral injection of VEGF also induce the degeneration of the dopaminergic system [77]. Another important consequence could be the infiltration of peripheral monocytes/macrophages, which could act as protector or neurotoxic [78]. These circumstances could increase the sensitivity of SN to inflammation.

## 6. Why Are the Dopaminergic Neurons of the SN Especially Affected in Our Inflammation Model?

We had described that dopaminergic neurons were especially sensitive to inflammation, and this did not occur when LPS was delivered within the MFB (dopaminergic axons) or the striatum (dopaminergic terminals). The main suggestion was that SN was highly vulnerable to oxidative damage [79–82], taking into account its reduced antioxidative capability along with its high content on iron and DA [83]. However, this rationale did not account for the special sensitivity of the DA neurons with respect to other non-DA neurons within the SN, such as the GABAergic ones. Although unexplained, this is very interesting for Parkinson's disease, in which the dopaminergic neurons are especially sensitive, probably not only due to the inflammatory process. 

**Figure 2:**
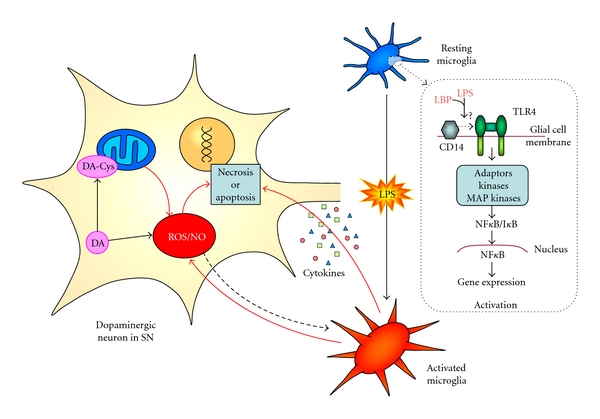
Interaction between glia and neurons may lead to neuron damage and death. LPS activates microglia from a resting state to an activated one, probably through the CD14 and the TLR4 receptors; this signalling pathway, mediated by different molecular adaptors, kinases, and MAP kinases, activates NF-*κ*B with the consequent transcription of specific genes leading to microglial activation. Then, activated microglial cells release several compounds, as proinflammatory cytokines, radical oxygen species (ROS), and NO that may eventually lead to neuronal death. Within dopaminergic neurons, the mitochondrial respiratory chain can be affected by several substances, leading to energy failure, production of ROS, and reduction of the neurons viability. ROS can act as signal for the activation of microglia, indicating that neurons are not healthy. DA can exert a toxic action through the ROS formed in its oxidative metabolism; it may also forms complexes with cysteine, inhibiting the respiratory chain and producing more ROS. The reduction/elimination of microglial induction could ameliorate neuronal damage.

To answer this question, we studied the possible influence of DA. There was a general agreement on the toxic capability of DA, which generates redox metabolites including semiquinone, quinone, zwitterionic 5,6-hydroxyindoles, and possibly oxygen free radicals. Theoretically, this neurotoxicity also accelerates autooxidation of the released DA, which results in the generation of free radicals *in vivo* (Figure 2). Enhanced DA autoxidation and oxygen free radicals may initiate a cascade of oxidant stress, leading to injury and loss of SNc neurons in Parkinson's disease. This possibility is supported by increased basal malonaldehyde formation (lipid peroxidation) and iron content in surviving pigmented SN neurons [80, 84, 85]. 

To test the involvement of DA in the degeneration induced by LPS, we treated albino Wistar rats with different concentrations of *α*-methyl-*p*-tyrosine (*α*-MPT), an inhibitor of TH activity that blocks synthesis of cytoplasmic DA [86, 87]. Pretreatment with *α*-MPT reduces DA content by 79% [88]. Results showed that *α*-MPT prevented, in a dose-dependent manner, the inflammatory features induced by LPS: activation of microglial cells (characterized by a change of morphology from resting, ramified cells with thin processes, to reactive microglial cells that eventually become phagocytic), loss of the astroglial population accompanied by the appearance of GFAP immunoreactive debris from hypertrophic, degenerating astrocytes, loss of TH immunostaining, and expression of mRNA for TH and DA transporter (DAT). These protective effects resulted from inhibition of TH and the consequent decrease in DA concentration, since cotreatment with L-DOPA/benserazide, which bypasses TH inhibition increasing the extracellular levels of DA in the SN approximately fourfold with respect to basal levels [89], avoided the protective effects induced by inhibition of TH produced by *α*-MPT and restored the degenerative effects induced by LPS on dopaminergic neurons as well as the loss of astrocytes. All these results strongly supported the implication of DA in the dopaminergic neurodegeneration induced by LPS in the SN. Moreover, not only did DA seem to be involved in dopaminergic degeneration of the SN but it could also be responsible for the special sensitivity of the SN to the inflammation induced by LPS. The SN inflammatory response to LPS injection after TH inhibition was similar to that described for other brain areas, where LPS had little or no effect on neuron degeneration or the glial inflammatory process [35, 36]. However, the treatment with LPS + *α*-MPT + L-DOPA/benserazide did not produce activation of microglial cells, although it induced damage to astroglia. This result is quite intriguing and could be involved in the inflammation process in SN. These results point out the important contribution of DA to the vulnerability and degeneration of dopaminergic neurons of the SN. Further knowledge about the involvement of DA in this process may lead to the possibility of new protection strategies against this important degenerative process.

## 7. Other Natural and Endogenous Compounds Able to Induce the Inflammatory Process along with the Degeneration of Dopaminergic Neurons by Intranigral Injection

LPS is a compound derived from de bacterial cell wall; thus, it could be considered an exogenous substance to the nervous system; other natural and endogenous compounds have been described to have similar properties on this process.

### 7.1. Histamine

We have evaluated the effects of a direct infusion of histamine (HA) in SN, striatum, medial septum, and medial lemniscus. There were many reasons for carrying out these experiments. Histamine is an important component of mast cells of the brain and participates in the enhancement of microvascular permeability, leukocyte rolling, adhesion, and extravasations of inflammatory cells into the brain and spinal cord. These events are important in many inflammatory diseases of the CNS, such as encephalomyelitis and multiple sclerosis [90]. It is also released after trauma, ischemia, seizures, and inflammation [91]. Moreover, the brain also has histaminergic neurons located in the tuberomammillary nucleus of the posterior hypothalamus nucleus that project to almost all regions of the brain from the olfactory bulb to the spinal cord [92]. This neuronal HA acts as neuromodulator or neurotransmitter [93, 94], participating also in many physiological functions [93–95]. It also interacts with some neurotransmitter as DA [96–101]. This relationship could be involved in the interaction between central HA neurons with the extrapyramidal system. It is known that HA induces catalepsy, which has been suggested as an animal model of Parkinson's disease [102]. HA antagonists are used as antiparkinsonian drugs [103]. 

HA has been shown to be involved in neuronal degeneration [104] and neurotoxicity [105]. Wernicke's encephalopathy is a disorder characterized by selective pathologic damage to the midline thalamus, mammillary bodies, and certain brainstem nuclei. Thiamine deficiency is a critical factor in the etiology of this disorder. Langlais et al. [104] using a rat model of Wernicke's encephalopathy induced by an acute bout of pyrithiamine-induced thiamine deficiency (PTD), pointed out that HA mediated neuronal death in this model.

In our study [106], the injection of histamine in SN produced an acute inflammatory response manifested by an activation of microglia, where OX-42-immunoreactive microglia exhibited typical features of phagocytic cells. In addition, a marked induction of MHC class II antigen expression and a characteristic disappearance of the GFAP immunoreactivity were produced in the same area. These effects were accompanied by a selective damage in dopaminergic neurons, evidenced by the loss of tyrosine hydroxylase mRNA-expressing cells, tyrosine hydroxylase-immunolabeled-positive cell bodies, and a decrease of DA and 3,4-dihydroxyphenylacetic acid levels. However, HA injection had little effect in other areas; only high doses produced an evident dopaminergic terminal degeneration. Moreover, similarly to that described for LPS, HA produced the specific degeneration of DA neurons of SN; HA did not affect GABAergic neurons, alter the pattern of ChAT mRNA-expressing cells, or change the pattern of 5-HT-immunolabeled cells when it was injected in SN, septum, or the medial lemniscus, respectively. We conclude that, similarly to LPS, HA is a selective neurotoxin for dopaminergic cells.

HA effect must be related to its inflammatory capability along with the rapid and reversible increase in the BBB permeability, producing a disturbance of the normal brain homeostasis. Moreover, a direct neurotoxic effect of HA over the nigral dopaminergic population should never be discarded, since there are reports on the induction of neuronal death by HA [104, 105]. In spite of the strict and remarkable selective neurotoxic action of HA on the dopaminergic system, HA may be taken into account in relationship to its involvement in the etiopathology of PD. So, higher blood HA levels have been found in patients with PD than in healthy controls [107]. However, central histaminergic systems are not affected in PD [108]. It has been shown that mast cells can rapidly penetrate brain blood vessels from extraneuronal sources [109]. It is interesting to note that therapies used in PD (L-DOPA + carbidopa) decrease the blood HA concentration [107], probably due to the decrease in its formation [110]. In addition, HA antagonists have long been used as antiparkinsonian drugs [103]. Patients with idiopathic PD treated with famotidine, an HA H2 antagonist, demonstrated improvement in their motor symptoms [111]. In addition, some HA metabolites and derivatives have been correlated with the degree of severity in PD patients. Levels of pros-methylimidazolacetic acid in cerebrospinal fluid showed a high positive correlation with the severity of PD in a group of not medicated, mildly to moderately affected patients [112, 113]. Further indications supporting the involvement of HA in the etiopathology of PD come from the observation that an increase in HA concentration was associated with the appearance or worsening of parkinsonian symptoms. Thus, acute tacrine administration produced tremulous jaw movements that shared some characteristics with parkinsonian tremor [114–116]. Tacrine is an inhibitor of brain histamine *N*-methyltransferase, which increases brain HA concentration [117]. Combined treatment with tacrine and haloperidol increases the parkinsonian-like symptoms [118, 119]. Secondary Parkinsonism has been described in neurocysticercosis [120, 121], characterized by a diffuse brain edema [122] generally accompanied by an increase of HA levels.

In conclusion, we showed for the first time that HA was able to produce a specific degeneration of dopaminergic neurons in SN along with a highly inflammatory process. The dose of HA needed to produce this effect was remarkably lower than that described in other studies. Besides, many of the relationships between elevated HA concentration and parkinsonism-like symptoms were pointed out. All these data strongly suggested an important relationship between elevated concentrations of HA and degeneration of the nigrostriatal dopaminergic system, which requires further investigation.

### 7.2. Thrombin

Thrombin is a multifunctional serine protease, best known for its role in the blood coagulation cascade. It is derived from its zymogen, prothrombin, and converts fibrinogen into fibrin. It has many functions: activates platelets, stimulating the proliferation of vascular smooth muscle cells [123–125]; it is involved in the chemotaxis and adhesion in monocytes, macrophages, and neutrophils (for review, see Grand et al. [126]); it has specific effects in cells of the CNS, regulating neurite outgrowth in neuronal cells [127] and inducing the proliferation of astrocytes [128]; it is also able to induce the activation and proliferation of cultured rodent microglia along with the production of nitric oxide (NO); it evokes the release of cytokines and chemokines [129] and induces the proliferation of astrocytes [128, 130]. The latter effects could be important in relationship to inflammatory processes. It is also interesting that brain contains this and some other proteases, along with protease-activated receptors (PARs) that serve key roles in blood coagulation and wound healing. These proteases have actually been found in the brain in situations in which the BBB is compromised, with a possible role in the neuropathological triggering of this situation (for review, see Gingrich and Traynelis [131]). Taking into account all these properties, along with the possible effect of thrombin in stroke, we studied thrombin effects on the nigrostriatal dopaminergic system.

Following the experimental procedure carried out in our previous works, we injected different amounts of thrombin into the nigrostriatal pathway [132]. Seven days after injection, we found a strong inflammatory response in the SN with a strong macrophage/microglial reaction pointed out by immunostaining (using OX-42 and OX-6 antibodies) and the induction of iNOS, IL-1*α*, Il-1*β*, and TNF-*α*. These results showed that the infusion of thrombin induced an inflammatory process in a similar way to other proinflammatory compounds such as LPS, probably by the activation of microglial cells, as described for microglial cultures [129]. The thrombin-induced inflammation was produced by its biological activity, maybe through the protease-activated receptors- (PAR-) receptor, since the injection of heat denatured thrombin or thrombin plus *α*-NAPAP (a specific inhibitor of thrombin) did not induce these processes, or they were strongly reduced. Moreover, selective damage to dopaminergic neurons was produced after thrombin injection, evidenced by loss of TH immunostaining and TH mRNA expressing cell bodies, and the unaltered transcription of glutamic acid decarboxylase (GAD) mRNA in the SN and striatum. These effects were also produced by its biological activity since they almost disappeared when thrombin was heat-inactivated or injected with its inhibitor *α*-NAPAP. 

These thrombin effects were even more interesting if we remember that thrombin is activated from prothrombin in the coagulation cascade and also that it enters interstitial fluid during penetrating head wounds, hemorrhagic stroke, rupture of cerebral aneurysms, and arteriovenous malformations. In addition, BBB breakdown associated with cerebrovascular insults reflects a largely nonselective increase in the permeability of brain capillaries and tight junctions to high molecular weight proteins. Moreover, taking into account that prothrombin circulates in blood at high concentrations (1 *μ*M) [133] and vascular injury triggers its rapid conversion to thrombin, direct entry of thrombin into the interstitial space with the consequence of a significant increase in its concentration is plausible [134]. Moreover, the elevation of thrombin levels in cerebrospinal fluid from 100 pM to 25 nM for a period of more than a week has been reported in subdural hematoma [135]. This suggests that appreciable amounts of thrombin can be generated and persist at sites of cerebrovascular injury. When bleeding occurs directly within brain tissue, active thrombin, and other proteases will freely penetrate the interneuronal spaces by diffusion until clotting closes the injured vessels and thrombin becomes depleted from the clot. High concentrations of thrombin, such as those that can be produced by a cerebral hemorrhage, appear to cause brain damage [133, 136] and may also contribute to damage in Alzheimer's disease and vascular dementia [137–139]. Vascular Parkinsonism has also been reported [140]; moreover, features of Parkinsonism have been seen in up to 38% of lacunar stroke patients at one year [141]. 

All these data provided evidence that thrombin activates microglia along with other inflammatory features in the SN, resulting in specific degeneration of dopaminergic neurons. These results suggest that thrombin, as an endogenous neurotoxin, could be implicated in dopaminergic neuron degeneration. Besides PD, these features could also be of relevance for a wide range of neurological diseases which present pathological signs of inflammation, such as cerebrovascular diseases and CNS trauma.

Similar results were described by Choi et al. [142]. It is interesting to note that the inflammatory process and the degenerative induction produced by intranigral injection of thrombin are not overcome by the treatment with dexamethasone as anti-inflammatory agent [143]. In fact, when we studied the possible protection exerted by dexamethasone in the thrombin paradigm, we found unexpected results. Dexamethasone was able to prevent partially the loss of astrocytes but was unable to overcome other inflammatory features, as the microglial proliferation induced by thrombin. Moreover, this treatment resulted in a greater loss of dopaminergic neurons that was accompanied by the accumulation of *α*-synuclein in dopaminergic neurons, probably produced by the diminution of its degradation, an effect described to be produced by dexamethasone on the beta-amyloid [144]. Dexamethasone produced other effects, as the consequent aggravation of the ischemic neuronal damage in striatum [145] and hippocampal CA1 area [146] as the extracellular accumulation of glutamate increased and thereby the overload of cellular calcium [147]. 

We also found that dexamethasone produced a significant decrease in PAR-1, which was increased by thrombin plus dexamethasone; nexin-1, the most abundant, potent endogenous thrombin inhibitor in brain [148], was not also affected by dexamethasone alone but was increased by thrombin; the effect was greater and in the same direction when thrombin and dexamethasone were used together. Dexamethasone produced a greater amount of oxidative stress (measured as carbonyl groups) than thrombin, in a similar extension that in animals treated with thrombin plus dexamethasone. We suggest that this increase of oxidative stress could be the cause of the great damage produced by dexamethasone. The cause of the oxidative stress is the increase of DA metabolism produced by the release of DA along with its metabolism through MAO enzymes. It is known that MAO-A is induced by glucocorticoids in tissues as human skeletal muscle [149] or bovine adrenal endothelial cells [150]. Dexamethasone increased the expression of MAO-B in rat astrocytes, which was inhibited by the glucocorticoids receptor inhibitor RU486 [151]. Moreover, the relationship between MAO-dependent H_2_O_2_ overproduction and degenerative processes had been suggested in PD [152]. These data suggested that H_2_O_2_ produced by MAO played an important role in the induction of apoptosis under some conditions. In our experimental design, both MAO A and B increased in animals treated with dexamethasone. Moreover, when we blocked MAO activity with tranylcypromine, the death of dopaminergic neurons caused by the combined treatment with thrombin and dexamethasone was prevented. This supports that induction of MAO activity by dexamethasone might play an important role in the lack of protection against thrombin observed in the animals treated with this glucocorticoid. This result is also in agreement with Tazik et al. [153], who recently described the protective effect of rasagiline, an inhibitor of MAO B, on dexamethasone-induced brain apoptosis. Increase of DA metabolism produced by dexamethasone through the induction of MAOs could be important in dopaminergic neurons, since DA seems to play an active role in their degeneration [154].

### 7.3. Tissue Plasminogen Activator (tPA)

tPA is a highly specific serine proteinase and one of the two principal plasminogen activators. It is widely distributed in all brain regions across the neuroaxis, being more elevated in the spinal cord and the SN [155, 156]. Different functions have been suggested for tPA in the CNS; for our work, the most important was the neurotoxicity effect [157–162]. Different animal models had demonstrated that the genetic deficiency of tPA [159, 163] and its inhibition with the natural tPA inhibitor neuroserpin [164, 165] were associated with a decrease in infarct volume and significant neuronal survival compared with control animals. Moreover, tPA plays important roles in blood coagulation and fibrinolysis outside the CNS. By cleaving the precursor molecule plasminogen, plasminogen activators produce the active enzyme plasmin, which dissolves fibrin-based clots in focal cerebral ischemia. Clinical trials have demonstrated that treatment with plasminogen activators in selected patients can improve outcome after acute ischemic stroke [166]. Moreover, tPA is the only drug approved for the treatment of thromboembolic stroke, but it might lead to some neurotoxic side effects. Some of these effects could be related with the fact that tPA, as thrombin, is able to induce microglia [167], probably with the consequent activation of an inflammatory process that could be responsible for some of the neurotoxic effects reported after its pharmacological use.

We have used the intranigral injection of tPA, which let us to describe its effects under standardized conditions. We found that injection in the SN led to the induction of inflammatory process, microglial activation, loss of astroglia, and increase in the expression of nNOS, iNOS, and aquaporin 4, along with the disruption of the BBB integrity. This latter result is in agreement with Yepes et al. [168] and Goto et al. [169] which showed that recombinant tPA promotes acute direct neurotoxicity with disruption of the BBB and increased formation of edema. Moreover, we also found a dose-dependent loss of dopaminergic neurons, pointed out by TH-immunoreactivity and changes in the expression of TH and DAT mRNAs. The toxic effect of tPA seemed to be specific on dopaminergic neurons of the SN since the GABAergic population of the ventral midbrain was not affected. 

These results could be related to—and could also justify—the possible neurotoxic effect produced by tPA in those pathological conditions which result in a significant increase of its production and release. It is known that endogenous tPA activity increases significantly by ischemia [159]; this effect is prevented in animals with genetic deficiency of tPA [159, 163] and also by the treatment with the natural tPA-inhibitor neuroserpin [164, 165]. Ischemia should be produced early in the SN to observe the degeneration of dopaminergic neurons induced for tPA. However, we must note that we carried out other experiments in which we injected tPA intravenously at nearly clinical concentration, along with the injection of saline solution in the SN in order to disrupt the BBB; we did not find a significant degeneration of the dopaminergic system, suggesting that the damage observed in our model should be produced by the increase in the endogenous concentration of tPA and its extravasations by isquemia.

### 7.4. Trisialoganglioside

 The intranigral injection of Trisialoganglioside GT1b, one of the major brain gangliosides, induced a great inflammatory response along with the degeneration of dopaminergic neurons [170]. This is also relevant since gangliosides, a component of membranes, are also involved in some neurodegenerative processes in which they accumulate. The ability of these compounds to induce inflammation could be important.

## 8. Peripheral Inflammation Enhanced the Inflammation Produced by the Intranigral Injection of LPS and, Consequently, the Degeneration of Dopaminergic Neurons

As we have described above, evidence suggesting that inflammation may play a central role in the cell loss seen in PD has been accumulating during years (see [14]; for review see [15, 16]). Moreover, some relationship between central inflammation and peripheral could be possible. It is known that the chance of developing AD is increased twice in aged persons exposed to systemic infections [171]. Furthermore, Strang [172] described the increased prevalence of peptic ulcer prodromal to idiopathic Parkinsonism [173]. Thus, we were interested in using our animal model of PD to study the implication of peripheral inflammation [174]. To achieve peripheral inflammation, we used a model of ulcerative colitis (UC) induced by dextran sulphate sodium (DSS) ingestion [175]. We described an increase in the levels of inflammatory markers from serum (TNF-*α*, IL-1*β*, IL-6, and the acute phase protein C-reactive protein). Moreover, it also increased the inflammatory parameters in SN (TNF-*α*, IL-1*β*, IL-6, iNOS, intercellular adhesion molecule-1 (ICAM-1), and microglial and astroglial populations) caused by the intranigral injection of vehicle in animals with UC. Consequently, peripheral inflammation induced by UC increased all the LPS-induced inflammatory markers examined in the ventral mesencephalon, demonstrating that SN becomes more sensitive to central inflammatory stimulus under conditions of peripheral inflammation. The inflammatory response of SN was associated to increased vulnerability of its dopaminergic population. These results suggest that inflammation produced in a peripheral organ (intestine) could induce loss of dopaminergic neurons in the SN, enhancing the inflammation and the dopaminergic degeneration induced in the SN by a previous inflammation (LPS). Moreover, we also studied the possibility that leukocyte infiltration through an impaired BBB could be involved in the deleterious effects of systemic inflammation on nigral dopaminergic population under conditions of brain inflammation. Brochard et al. [176] had shown lymphocyte T infiltration into brain parenchyma in MPTP-intoxicated mice showing induction of ICAM-1 but no BBB disruption. Our analysis demonstrated that UC induced ICAM-1 levels in the ventral mesencephalon; flow cytometry analyses showed that the amount of circulating monocytes was higher in animals from the LPS and UC groups (around fourfold) than in the control animals. Infiltration in the UC + LPS group increased nearly eightfold. Moreover, using the intravenous injection of clodronate encapsulated in liposomes (ClodLip), which produced a peripheral macrophage depletion lasting 5 days in blood, liver and spleen of normal rats and mice [177–179], we found the reversion of the deleterious effect of peripheral inflammation on microglial activation, BBB disruption, astrocytes loss, and degeneration of nigral dopaminergic neurons induced by LPS. Taken together, our results demonstrate that peripheral inflammation induced by UC contributes to dopaminergic degeneration. Activation of macrophages seems a decisive factor, since destruction of this peripheral leukocyte type by ClodLip abolishes the nocive effects on the ventral mesencephalon associated with UC. This study shows that BBB disruption may increase brain susceptibility to subsequent exposure to circulating leukocytes. This study may also shed light into previous observations; it described the existence of an increased prevalence of peptic ulcer prodromal to idiopathic Parkinsonism [172, 180]. This has prompted some authors to suggest a prominent role of inflammation in gastrointestinal tract in the etiology and pathogenesis of idiopathic Parkinsonism, including a possible role for *Helicobacter pylori* infections [173, 181]. This infective process is the most prevalent in the world, affecting approximately 50% of the population [182], and it is considered the causative agent of many gastrointestinal and extradigestive conditions. Colonization of gastric mucosa by *H. pylori* is accompanied by an inflammatory response associated with gastric mucosal damage through the activation of polymorphonuclear neutrophil leukocytes [183], inflammatory infiltration of lymphocytes, plasma cells and macrophages in the stomach tissue [184–186], and the production of pro-inflammatory factors such as IL-8, IL-1, and TNF-*α* [187–189].

## 9. Conclusions

On 1998, we proposed an animal model of PD based on the inflammatory process triggered by the intranigral injection of LPS. Afterwards, this model was further developed and corroborated that the SN had the highest response to inflammation process induced by LPS with respect to other brain areas studied. In addition, one of the responses to inflammation was the specific degeneration of dopaminergic neurons. The implication of inflammation in the degeneration of dopaminergic neurons was also supported by the protection produced by many different anti-inflammatory compounds. The interest of this model is to point out the fact that inflammation is the inductor of the degeneration of dopaminergic neurons, probably in cases as injury, boxing, infection, and others; it could be important in the progression of the disease at the same time that it could enhance the damage induced by the main (unknown) cause of the disease. Moreover, the interest of the scientific community in inflammation in PD has increased significantly afterwards. In all cases, the anti-inflammatory pharmacology could be important in the disease, and this model could be also very useful to test anti-inflammatory treatments. Moreover, the model has allowed pointing out that any endogenous substance able to induced inflammation or activation of microglia produced similar effects to LPS. Many endogenous compounds have been used in this way, as histamine, thrombin, or tPA among others. Some of them could have a direct effect in this disease, as stroke or infection. With respect to the special sensitivity of the SN to inflammation, there are no clear answers, but its knowledge would be of interest for the treatment of PD. The special sensitivity of dopaminergic neurons to inflammation is also of special interest; in this case, however, the increase of free radicals production through MAO enzymes and the effect of DA seem clear. Our model make possible to study other processes, as peripheral inflammation, which could enhance the effects produced by central inflammation.
